# Comprehensive Genomic Discovery of Non-Coding Transcriptional Enhancers in the African Malaria Vector *Anopheles coluzzii*

**DOI:** 10.3389/fgene.2021.785934

**Published:** 2022-01-10

**Authors:** Inge Holm, Luisa Nardini, Adrien Pain, Emmanuel Bischoff, Cameron E. Anderson, Soumanaba Zongo, Wamdaogo M. Guelbeogo, N’Fale Sagnon, Daryl M. Gohl, Ronald J. Nowling, Kenneth D. Vernick, Michelle M. Riehle

**Affiliations:** ^1^ Institut Pasteur, Université de Paris, CNRS UMR 2000, Unit of Insect Vector Genetics and Genomics, Department of Parasites and Insect Vectors, Paris, France; ^2^ Institut Pasteur, Université de Paris, Hub de Bioinformatique et Biostatistique, Paris, France; ^3^ Department of Microbiology and Immunology, Medical College of Wisconsin, Milwaukee, WI, United States; ^4^ Centre National de Recherche et de Formation sur le Paludisme (CNRFP), Ministry of Health, Ouagadougou, Burkina Faso; ^5^ University of Minnesota Genomics Center, Minneapolis, MN, United States; ^6^ Department of Genetics, Cell Biology and Development, University of Minnesota, Minneapolis, MN, United States; ^7^ Department of Electrical Engineering and Computer Science, Milwaukee School of Engineering (MSOE), Milwaukee, WI, United States

**Keywords:** mosquito, STARR-seq, anopheles, non-coding, regulatory element, enhancer

## Abstract

Almost all regulation of gene expression in eukaryotic genomes is mediated by the action of distant non-coding transcriptional enhancers upon proximal gene promoters. Enhancer locations cannot be accurately predicted bioinformatically because of the absence of a defined sequence code, and thus functional assays are required for their direct detection. Here we used a massively parallel reporter assay, Self-Transcribing Active Regulatory Region sequencing (STARR-seq), to generate the first comprehensive genome-wide map of enhancers in *Anopheles coluzzii*, a major African malaria vector in the Gambiae species complex. The screen was carried out by transfecting reporter libraries created from the genomic DNA of 60 wild *A. coluzzii* from Burkina Faso into *A. coluzzii* 4a3A cells, in order to functionally query enhancer activity of the natural population within the homologous cellular context. We report a catalog of 3,288 active genomic enhancers that were significant across three biological replicates, 74% of them located in intergenic and intronic regions. The STARR-seq enhancer screen is chromatin-free and thus detects inherent activity of a comprehensive catalog of enhancers that may be restricted *in vivo* to specific cell types or developmental stages. Testing of a validation panel of enhancer candidates using manual luciferase assays confirmed enhancer function in 26 of 28 (93%) of the candidates over a wide dynamic range of activity from two to at least 16-fold activity above baseline. The enhancers occupy only 0.7% of the genome, and display distinct composition features. The enhancer compartment is significantly enriched for 15 transcription factor binding site signatures, and displays divergence for specific dinucleotide repeats, as compared to matched non-enhancer genomic controls. The genome-wide catalog of *A. coluzzii* enhancers is publicly available in a simple searchable graphic format. This enhancer catalogue will be valuable in linking genetic and phenotypic variation, in identifying regulatory elements that could be employed in vector manipulation, and in better targeting of chromosome editing to minimize extraneous regulation influences on the introduced sequences.

**Importance:** Understanding the role of the non-coding regulatory genome in complex disease phenotypes is essential, but even in well-characterized model organisms, identification of regulatory regions within the vast non-coding genome remains a challenge. We used a large-scale assay to generate a genome wide map of transcriptional enhancers. Such a catalogue for the important malaria vector, *Anopheles coluzzii*, will be an important research tool as the role of non-coding regulatory variation in differential susceptibility to malaria infection is explored and as a public resource for research on this important insect vector of disease.

## Introduction

Transcriptional enhancers are non-coding *cis*-regulatory elements that are responsible for most of the regulated gene expression in eukaryotic genomes. Promoters, located just upstream of transcription start sites, mediate only basal levels of unregulated gene expression. Enhancers bind transcription and other protein factors and subsequently interact with physically distant promoters. This enhancer-promoter interaction drives the majority of regulated gene expression of enhancer target genes (Robson et al., 2019; Schoenfelder and Fraser, 2019; Andersson and Sandelin, 2020).

Genetic variation in enhancer sequences can differentially influence gene expression and appears to underlie complex medically and agriculturally important phenotypes (Bird et al., 2006; Hrdlickova et al., 2014; Gloss and Dinger, 2018; Zheng et al., 2019). Indeed, genetic variation in coding regions of the genome have been shown to explain little of the variation in phenotype. Results from genetic mapping methods such as genome wide association surveys (GWAS) indicate that at least 90% of significant GWAS candidate variants in humans are found within the non-coding genome, and most of these significant variants appear to be located within enhancers (Neph et al., 2012; Schaub et al., 2012; Farh et al., 2015).

A challenge in identifying transcriptional enhancers is the absence of an amino acid-like code that could facilitate their accurate recognition. An additional obstacle to developing accurate computational algorithms for enhancer prediction is the paucity of experimental data from different systems. The low sequence complexity and repetitive nature of non-coding regions of the genome also make computational prediction more challenging (Kleftogiannis et al., 2016; Lim et al., 2018; Hong et al., 2021).

Transcriptional enhancers can be identified by either direct or indirect experimental approaches (Farley et al., 2021). Direct approaches measure enhancer activity of nucleotide sequence through assays such as manual luciferase reporter assays. A manual luciferase reporter assay queries the ability of a candidate sequence to regulate reporter gene transcription, which is quantified by luminescence produced by the luciferase protein product. Direct detection measures the inherent transcriptional regulatory activity of a given nucleotide sequence isolated from its dynamic chromatin context (Arnold et al., 2013; Arnold et al., 2014). In contrast, indirect approaches identify enhancers as correlates of genomic regions differing in their chromatin accessibility or histone modification markers. The indirect strategies such as ChIP-seq and ATAC-seq (Gomez-Diaz et al., 2017; Ruiz et al., 2019; Farley et al., 2021; Ruiz et al., 2021) infer enhancer presence based on chromatin properties such as chromatin accessibility and histone modifications, but do not directly or functionally test or confirm enhancer activity. Methods used to survey histone signatures and chromatin accessibility may also detect transcriptional silencers, insulators and other features in addition to enhancers, and the functional category of detected elements is not necessarily distinguishable without additional work, for example direct functional assays.

In order to understand the phenotypic significance of enhancers and their nucleotide variation, it is essential to be able to filter the genome for functional enhancer elements. Enhancers have typically been studied using manual plasmid reporter assays to query a cloned candidate enhancer fragment. However, such manual reporter assays are not suitable for genome-wide screening because each assay assesses the activity of a single enhancer candidate and they are not multiplexable or scalable to the genome level (Muerdter et al., 2015).

Here, Self-Transcribing Active Regulatory Region sequencing (STARR-seq) was used to generate a genome wide catalog of transcriptional enhancers in the malaria vector *Anopheles coluzzii*. STARR-seq is a massively parallel reporter assay that provides the scale necessary for genome-wide screening and activity measurement of transcriptional enhancers. The approach is essentially a highly multiplexed version of the standard plasmid-based manual luciferase reporter assay that measures the transcriptional regulatory activity of a cloned nucleotide sequence. Briefly, in the STARR-seq screen, randomly sheared candidate genomic fragments are cloned into the 3’ UTR of an irrelevant reporter gene in a plasmid with a basal promoter, resulting in the candidate sequence being transcribed as part of the reporter gene transcription unit. Candidate fragments that possess enhancer activity stimulate increased transcription from the basal promoter, which generates a transcript of the reporter gene as well as the inserted candidate enhancer sequence. Rather than measuring luciferase activity, here RNA-seq is used to detect, in a massively parallel way, all of the reporter transcripts generated from the cloned genomic library transfected into cells. Trimming the flanking reporter sequence from the transcripts yields the RNA-seq reads originating from the collection of genomic fragments that were cloned in the plasmid library, which are then mapped to the reference genome assembly. The RNA-seq reads, originally the randomly sheared genomic DNA library, tile across the genome, and the normalized counts of each mapped window as compared to the control reveals enrichment that defines genomic peaks indicating functional enhancers, as well as a quantitative measure of their level of enhancer activity by normalized sequence read counts.

This massively parallel screening approach evaluates the enhancer activity of nucleotide sequences removed from their native chromatin structure, and thus generates a comprehensive catalogue of enhancers that may be differentially active across different cell types and developmental times. STARR-seq has been implemented in *Drosophila melanogaster* (Arnold et al., 2014), plants (Benoit, 2020) and human (Arnold et al., 2013), but has not been used in mosquitoes. Complementary approaches examining chromatin accessibility including FAIRE-seq (Perez-Zamorano et al., 2017) and ATAC-seq (Ruiz et al., 2021) have been used in *Anopheles* mosquitoes, as well as experiments for computational prediction of enhancers (Asma and Halfon, 2019; Schember and Halfon, 2021).

A comprehensive understanding of mosquito regulatory biology and the regulation of gene expression will require a careful combination and cross validation of data generated using direct, indirect and *in silico* approaches. The 3,288 candidate enhancers identified in *A. coluzzii* will be valuable in linking genetic and phenotypic variation where efforts focused on the coding genome have not yielded answers, in finding new regulatory elements, and in informing the choice of target sites for chromosome editing while minimizing disruptive secondary effects upon gene regulation in the chromosomal domain. A greater understanding of mosquito regulatory networks will add important tools to the malaria vector control arsenal.

## Materials and Methods

### Source of Mosquito Genomic Deoxyribonucleic Acid and Generation of Input Genomic Library

Genomic libraries were prepared using a modified version of the methodology previously presented (Arnold et al., 2013). Mosquito DNA was derived from field material collected from two localities in Burkina Faso from 2007–2009 (Riehle et al., 2011). Samples were collected as larvae and reared to adulthood before being typed for species by the SINE200 × 6.1 assay (Santolamazza et al., 2008). DNA from 60 *A. coluzzii* were pooled (at equal volume) and sheared using a S220 Focused-ultrasonicator (Covaris) to produce fragments ∼800 bp-1kb.

After shearing, an Illumina TruSeq Nano DNA Library Prep Kit was used according to the manufacturer’s instructions to end-repair, A-tail, and prepare two independent sequencing libraries differing only in the sequence index tag using Illumina TruSeq indices 5 (ACAGTG) and 19 (GTGAAA). Enrichment of adapter-ligated molecules was carried out by PCR in order to add cloning adaptors as follows: an initial denaturation at 95°C for 5 min 10 cycles of 98°C for 15 , 60°C for 30 s, and 72°C for 30 s followed by a final extension of 72°C for 5 min. Each reaction was comprised of 5 ng DNA template, 10 uM 2.5 ul STARR_Seq_PCR1_For primer (10 uM), 2.5 ul STARR_Seq_PCR1_Rev primer (10 uM), 25 ul 2x KAPA ReadyMix (KAPA Biosystems) and nuclease-free H_2_O to a final reaction volume of 50ul. Primer sequences for this and all experimental procedures are provided in Supplementary Table S1. Amplification products were purified with AMPure XP beads (Beckman Coulter) and the libraries were quantified using a Quant-IT PicoGreen dsDNA assay (Thermo Fisher Scientific) and the resulting fragment size distribution was assessed using a Bioanalyzer (Agilent Technologies).

### Cloning and Transfection of Genomic Libraries

Libraries were cloned into the screening vector, pSTARR-seq_fly, kindly supplied by Alexander Stark (available from AddGene as pSTARR-seq_fly, vector #71499). Prior to cloning, the vector was linearized by digestion with SalI and AgeI for 5 h at 37°C, gel purified using the QIAquick Gel Extraction Kit (Qiagen), and further purified and concentrated using the QIAquick PCR Purification Kit and MinElute PCR Purification Kit respectively (Qiagen). Final elution was carried out in nuclease-free H_2_O.

In-Fusion Cloning was performed in a 10ul reaction containing 30 ng mosquito DNA (15 ng from each of the uniquely indexed library and 30 ng of linearized vector. In total, 54 In-Fusion reactions were prepared in order to ensure comprehensive cloning of the *A. coluzzii* genome. Reactions were ethanol precipitated in batches of 4-5 reactions, resuspended in nuclease-free H_2_O and pooled to prepare a final stock that was used for transformations into MegaX DH10B T1R Electrocomp Cells (Invitrogen). Cells were electroporated (2.0 kV, 200 Ω, 25 uF) in cooled Gene Pulser cuvettes with 0.1 cm electrode gap size using the Gene Pulser Xcell Electroporation System (Bio-Rad), and recovered in 1 ml recovery medium. After 1 h recovery at 37°C/220 rpm, 5 transformations were used to inoculate 500 ml LB with ampicillin (1ug/ml) and incubated at 37°C shaking at 220 rpm until the OD_600_ reached 0.8–1.0. plasmid libraries were extracted using the Plasmid Plus Mega Kit (Qiagen), pooled, and quantified. A total of 60 transformations were prepared in order to sufficiently capture the library.

### Pre- and Post-Cloning Sequencing

Library sequencing was carried out before and after cloning in order to ensure no loss of genome coverage due to a bottleneck in the cloning step. For the pre-cloning DNA, the following PCR reaction was set up to prepare the samples for sequencing: 5 ng template DNA, 2.5 ul Multiplexing PCR Primer 1.0 (10 uM, AAT​GAT​ACG​GCG​ACC​ACC​GAG​ATC​TAC​ACT​CTT​TCC​CTA​CAC​GAC​GCT​CTT​CCG​ATC​T), p7 primer (10uM, CAA​GCA​GAA​GAC​GGC​ATA​CGA), 25 ul 2x KAPA HiFi HotStart ReadyMix (KAPA Biosystems) and nuclease-free H_2_O to a final reaction volume of 50 ul. This reaction was amplified using the following PCR conditions: 95°C for5 min., 10 cycles of 98°C for15 s, 58°C for30 s, 72°C for 30 s. and a final extension of 72°C for 5 min.

For the post-cloning DNA, the following PCR reaction was set up to prepare the samples for sequencing: 32.5 ng of template DNA (this DNA amount reflects the same number of insert molecules as the reaction described above, but accommodates the pSTARR-seq_fly vector), 2.5 ul Multiplexing PCR primer 1.0 (10 uM), p7 primer (10 uM), 25 ul 2x KAPA HiFi HotStart ReadyMix (KAPA Biosystems) and nuclease-free H_2_0 to a final reaction volume of 50 ul. This reaction was amplified using the following PCR conditions: 95°C/5 min, 10 cycles of [98°C/15 s, 58°C/30 s, 72°C/30s] with a final extension at 72°C/5 min.

The PCR reactions were cleaned up with 1x AMPureXP beads, resuspended in 30 ul Elution Buffer (EB) (10 mM Tris-HCl, pH 8.5). The final pooled sample was quantified using a Quant-IT PicoGreen dsDNA assay (Thermo Fisher Scientific) and the resulting fragment size distribution was assessed using a Bioanalyzer (Agilent Technologies). The libraries were denatured and diluted according to Illumina’s guidelines and sequenced on the Illumina HiSeq 2,500 in high output mode using 2 × 125 bp reads.

### Cell Culture and Transfection With Cloned Genomic Library

The STARR-seq enhancer screen was carried out in three biological replicates, where each replicate is defined as an independent instance of the entire pipeline including transfection of *Anopheles* 4a3A cells (Muller et al., 1999), cDNA and plasmid isolation, and Illumina sequencing, each of these steps as described below. 4a3A cells were maintained on Insect X-Press media (Lonza) supplemented with 10% heat inactivated Fetal Bovine Serum, at 27°C. No antibiotics were used. Cells were species-typed by a molecular diagnostic assay (Santolamazza et al., 2008) and determined to be derived from *A. coluzzii*, which was not yet described as distinct from *A. gambiae* at the time the 4a3A cell line was established (Supplementary Figure S1).

Across the three replicates, a total of 4.8 × 10^8^ 4a3A cells were prepared for transfection using Lipofectamine 3,000 Reagent (Invitrogen). Of these, ∼4 × 10^8^ cells (40 transfections) were used for RNA extraction, and ∼8 × 10^7^ cells (8 transfections) were used for plasmid extraction for the input control. Cells were seeded (1 × 10^7^ cells/25 cm^2^ flask) on day 1, transfected with 10ug library after 24 h (day 2), and RNA or plasmid was extracted after an additional 24 h post transfection (day 3). The transfection solution was prepared as a master mix, where for one transfection, a ‘DNA mix’ (140ul Opti-MEM [Thermo Fisher Scientific] + 10 ug plasmid library +20 ul P3000) and ‘Lipofectamine 3,000 Mix’ (140 ul Opti-MEM + 10 ul Lipofectamine) were prepared separately. Once the mixes were combined, the master mix was incubated at room temperature for 15 min, and the full volume added to a flask of cells.

### RNA and Plasmid Isolation From Transfected Cells

Cells were scraped from the base of each flask. Cells from 10 flasks were pooled and pelleted by centrifugation (2000 g/2 min), washed in 1x phosphate buffered saline (PBS) and re-pelleted (2000 g/2 min). The supernatant was removed once more, and any excess PBS was aspirated from the pellet. Total RNA was extracted from the cell pellet using the RNeasy Midi Kit (Qiagen) with an on-column DNA digestion using the RNase-Free dnase Set (Qiagen). Isolation of mRNA was carried out using the Dynabeads mRNA Purification Kit following the standard protocol. The mRNA was eluted off the beads at 70°C for 2 min, concentration measured on a NanoDrop, and stored at −80°C following the addition of ribonuclease inhibitor, RNasin [40 U/ul] (Promega). Using cells collected in the same manner, plasmid DNA was isolated using the Plasmid Plus Midi or Mini Kits (Qiagen). Plasmid extractions were quantified and stored at −20°C.

### Reverse Transcription

Reverse transcription was performed using SuperScript IV First-Strand cDNA Synthesis System (Invitrogen) on 5 ug mRNA per replicate. These reactions were carried out using 400 ng mRNA/reaction according to supplier instructions with the following modifications: a construct specific primer was used (RT_Rev, Supplementary Table S1, as described in (Arnold et al., 2013) and reactions (transcription reaction mix plus annealed RNA-primer mix) were incubated at 50°C for 10 min after which RNA was removed by the simultaneous addition of 1ul rnase H (2 U/ul) and 1 ul rnase A (10 mg/ml) (Thermo Scientific) per reaction, incubated at 37°C for 30 min. Finally, the cDNA was cleaned using QIAQuick PCR Purification Kit (Qiagen) and eluted in Elution Buffer (EB).

### cDNA and Input Control Plasmid Amplification and Sequencing

Primers used for specific amplification of cDNA, Report_Fwd and Report_Rev, are as in (Arnold et al., 2013) and also in Supplementary Table S1. cDNA from each sample was amplified for sequencing in four separate PCR reactions containing 35 ng template each, according to the method of (Arnold et al., 2013). The following reaction composition was used for the cDNA amplifications included 35 ng template DNA, 25ul KAPA HiFi HotStart ReadyMix (KAPA Biosystems), 0.25 ul Report_Fwd primer (100uM), 0.25 ul Report_Rev primer (100uM). These reactions were amplified using the following PCR conditions: 98°C/45 s, 15 cycles of [98°C/15 s, 65°C/30 s, 72°C/70 s]. Next, the PCR reactions were cleaned up with 1x AMPureXP beads and resuspended in 20 ul EB (10 mM Tris-HCl, pH 8.5), and a second PCR was performed to generate the final sequencing libraries. For each of the four reactions per sample, all 20ul of template from the cleaned-up initial cDNA PCR was used to set up the following PCR reactions: 20 ul DNA template, 25 ul KAPA HiFi HotStart ReadyMix (KAPA Biosystems), 2.5 ul D50X forward indexing primer (10 uM), 2.5 ul p7 primer (10 uM). The reactions were amplified using the following PCR conditions: 95°C/5 min, 10 cycles of [98°C/15 s (sec.), 58°C/30 s, 72°C/30 s] with a final extension at 72°C/5 min. The four reactions per sample were pooled, cleaned up with 1x AMPureXP beads, resuspended in 30 ul EB (10 mM Tris-HCl, pH 8.5). The final pooled sample was quantified using a Quant-IT PicoGreen dsDNA assay (Thermo Fisher Scientific) and the resulting fragment size distribution was assessed using a Bioanalyzer (Agilent Technologies). The libraries were denatured and diluted according to Illumina’s guidelines and sequenced on the Illumina HiSeq 2,500 in high output mode using 2 × 125 bp reads.

Plasmid control samples were amplified and sequenced in the same manner as the cDNA samples described above with the exception that the primers used for specific amplification of the plasmid template were Plasmid_Fwd and Plasmid_Rev, (Arnold et al., 2013), and listed in Supplementary Table S1.

### Sequence Analysis, Enhancer Peak Calling and Enrichment

The quality of sequencing reads was tested using FastQC version 0.11.5 (Wingett and Andrews, 2018). Following quality control, reads were mapped against the *Anopheles gambiae* AgamP4 reference genome assembly using BWA MEM (Li and Durbin, 2009) (version 0.7.7) with default parameters. Samtools version 1.6 (Li et al., 2009; Danecek et al., 2021) was used to select only the properly mapped reads (option -f 2), to filter supplementary alignments (option -F 2048) and to convert the sam files to bam files.

Prior to peak calling, reads from sequencing libraries run across multiple sequencing runs were merged and duplicate reads were removed from merged libraries, resulting in one sequencing library per biological replicate, which was then subjected to peak detection. Peak calling to detect significant enrichment in cDNA reads as compared to plasmid DNA reads was performed with R version 3.4.1 (R Core Team, 2016) using the package BasicSTARRseq version 1.4.0 (Buerger, 2015) with the function getPeaks and the following parameters: minQuantile = 0.99, peakWidth = 500, maxPval = 0.001, deduplicate = F and model = 2. For this step, reads mapped to the Y, UNKN and mitochondrial chromosomes were not analyzed. Only peaks displaying sequence read enrichment (cDNA reads/plasmid DNA reads) ≥ 3 were retained (Supplementary Figure S2). The common peaks across the three biological replicates were called with the function findOverlaps from the GenomicRanges package (Lawrence et al., 2013) with a minimal overlap set to 250.

### Genome Location of Enhancer Peaks

For each of the 3,288 enhancers, UTR, exon and mRNA scores corresponding to the fraction of the enhancer overlapping each genomic feature were calculated using bedtools intersect (Quinlan and Hall, 2010) (v 2.26.0). For enhancers overlapping multiple features of the same type (e.g., two different exons), only the larger score was retained. Then, the bedtools closest tool was used to determine the gene in closest proximity to the left and right of the candidate enhancer. A distance equal to zero indicates an overlap between the gene and the enhancer. Enhancers with no overlap with an annotated gene were classified as “intergenic” while those overlapping a UTR region or a CDS feature (intron or exon) were assigned to the corresponding category (enhancers overlapping both features, CDS and UTR, were assigned to the feature with the higher score). For enhancers overlapping the mRNA feature but no CDS or UTR features, bedtools closest was used to determine the intron in which they are located.

### Candidate Validation by Manual Luciferase Reporter Assays

PCR amplicons spanning candidate enhancer regions were amplified from the DNA pool of 60 *A. coluzzii* mosquitoes used for construction of the library. Resulting PCR fragments were cloned into the pCR8/GW/TOPO vector (Invitrogen) The PCR fragments were Gateway cloned into pGL-Gateway-DSCP, kindly supplied by Alexander Stark (available at AddGene, vector # 71,506) using Gateway LR clonase II (Invitrogen) as per the manual. Gateway clones were transformed into OneShot OmniMax 2T1 Phage-Resistant Cells, grown up overnight, plasmid purified (PureLink Quick Plasmid Miniprep Kit, Invitrogen) and sequenced with the primers LucNrev 5′ CCT​TAT​GCA​GTT​GCT​CTC​C and RVprimer3 5’ CTA​GCA​AAA​TAG​GCT​GTC​CC to verify the insert sequence. Primers used for amplification of candidate enhancers and negative control fragments are available in Supplementary Table S1.

Transfections were prepared in 96 well plates. *A. coluzzii* 4a3A hemocyte-like insect cells were seeded at 1 × 10^5^cells/well, in a total of 65ul with growth media. Cells were gently agitated on a MixMate (Eppendorf) for 30 s at 350 rpm to ensure even distribution and incubated for 24 h at 27°C. Transfections were carried out using Lipofectamine 3,000 (Invitrogen) and 2 vectors for dual luciferase assays: a renilla control vector pRL-ubi-63E (AddGene, #74280), and the pGL-Gateway-DSCP (described above) carrying a single amplified haplotype of the candidate enhancer upstream of a firefly luciferase gene. Renilla and firefly plasmids were transfected at a ratio of 1:5 (renilla:firefly) which equated to 18 ng renilla and 90 ng luciferase vector in 10ul volume per well. Plates were agitated for 30 s at 350 rpm on a MixMate (Eppendorf) to ensure mixing and incubated for 24 h at 27°C. The Dual-Glo luciferase Assay System (Promega) was used for luciferase assays, according to supplier instructions. Measurements were recorded on the GloMax Discover (Promega) at 25°C, with two 20 min incubations, one after the addition of Dual-Glo luciferase reagent and another after the addition of Stop & Glo reagent. All test plates contained a previously reported negative control fragment which was a size matched fragment within intron 1 of AGAP007058, and a highly active positive control enhancer peak nearby in AGAP008980 (Nardini et al., 2019). All samples were run in 6-fold replication within a single plate and across at least two independent plates for at least two biological replicates. In order to eliminate any effects of evaporation, only the 60 internal wells of the plate were used for transfection and measurement of luciferase activity. The external 36 wells were filled with cell media to a volume matching the internal wells. Firefly luciferase measurements (relative light units, RLUs) were corrected against the renilla measurements for the same well. These measurements were then normalized to the firefly/renilla mean for the negative control on the same plate to combine results across replicates. Luciferase activity was compared using a non-parametric ANOVA (Kruskal-Wallis) with post hoc pairwise comparisons.

To test the enhancer activity in multiple cellular backgrounds, a subset of enhancers was screened for activity in Ag55 cells, an *A. coluzzii* first-instar larval cell line (Pudney et al., 1979). Like the 4a3A cell line, the Ag55 line was originally described as *A. gambiae*, but molecular diagnostics confirm that, like 4a3A, it is also derived from *A. coluzzii* (Supplementary Figure S1). Cells were grown as described previously (Hire et al., 2015) and luciferase assays were performed as described above. Relative light unit output was compared between 4a3A and Ag55 cells using 2-way ANOVA followed by pairwise comparison.

### Genomic Sequence Characteristics of Candidate Enhancers

All 3,288 candidate enhancers were analyzed computationally for underlying sequence composition. For each candidate enhancer peak a matching control sequence was chosen by randomly selecting a window of the same size within 0.5 Mb on the same chromosome. When the randomly chosen window overlapped with either an exon or a candidate enhancer peak or when >1% of the sequence were unknown nucleotides (N’s), a new random sample was generated. This process was repeated 10 times for each peak, resulting in 10 control data sets, each containing 3,288 genome wide control fragments.

GC Content. GC content was calculated for each sequence in the control and candidate enhancer sets. Distributions of the GC content were compared across the control sets using a Kruskal-Wallis. If the 10 sets of control sequences were not statistically distinguishable at *p* < 0.001, a second Kruskal-Wallis test was run on the 10 control sets plus the treatment set (11 groups total) using the same significance level. If a statistically significant difference was observed, pair-wise post-hoc tests between each control set and the treatment set were performed with non-parametric Mann-Whitney U tests at *p* < 0.001. Finally, the GC content was considered significantly different if all 10 post-hoc tests were statistically significant and all controls were changed in the same direction (all increased or all decreased) relative to the candidate enhancers.

Perfect Repeat Analysis. Candidate enhancers and control fragments were analyzed for the presence of perfect mononucleotide (e.g., AAAAAA), dinucleotide (e.g., ATATAT), and trinucleotide (e.g., ATCATC) repeats. Repeats were required to be ≥ 6 nucleotides in length; 6 repeat units in the case of a mononucleotide repeat, 3 for a dinucleotide repeat and 2 for a trinucleotide repeat. Mononucleotide repeats were excluded from analyses of dinucleotide and trinucleotide repeats and dinucleotide repeats excluded from the analysis of trinucleotide repeats. The analysis was performed in two stages. First the fraction of the 3,288 sequences containing each type of repeat was examined. For each sequence repeat (e.g., TTTTTT or ACACAC), ten sets of control fragments were also examined. Kruskal-Wallis tests were used to compare perfect repeat presence across the 10 sets of control fragments. If the 10 groups of control fragments were not statistically distinguishable at *p* < 0.001, an additional Kruskal Wallis test was run using the 10 control sets and the candidate enhancer set (11 groups in total) with the same significant level. It the Kruskal Wallis test detected a significant difference among the 11 groups, pair-wise post-hoc tests between each control set and the candidate enhancer set were performed with non-parametric Mann-Whitney U tests as *p* < 0.001. Finally, a given repetitive element was considered significantly different if all 10 post-hoc tests were statistically significant and all control averages were changes in the same direction (all increased or all decreased) relative to the candidate enhancer average.

Secondly, for each specific repeat, control and treatment sequences containing the specific repeat were further analyzed examining repeat counts (the number of repeat occurrences per fragment), repeat length distribution, and the total fraction of the sequence involved in the repeat. The same statistical testing and filtering criteria describe above for repeat presence were applied in the examination of these attributes.

Enrichment of the four dinucleotide repeats CA, GA, GC, and TA and their reverse complements were calculated for candidate enhancers and the non-coding control sequences. GC and TA repeats are their own reverse complements and were counted twice. Enrichment of the dinucleotide repeat was calculated as log_2_ (fraction of enhancer sequences containing the dinucleotide perfect repeat/fraction of control sequences containing the same dinucleotide perfect repeat). As with work done in fruit flies and humans, these repeats needed to be at least 6bp in length. The analysis was repeated 10 times (once against each control) and results used to calculate means and standard deviations.

Transcription Factor Binding Site Motifs. Transcription factor binding sites (TFBS) were identified using 143 JASPAR 2020 core insect TFBS motifs from *D. melanogaster* (Fornes et al., 2020). Motifs were identified using the default paraments in AME (McLeay and Bailey, 2010) from MEME Suite (Bailey et al., 2015). The treatment sequences were tested against each set of control sequences for a total of 10 independent runs. The ten independent comparisons were assessed and only those motifs enriched in at least 8 of the 10 AME comparisons of candidate enhancers with controls were considered enriched.

In addition to comparing to known TFBS motifs from *D. melanogaster*, we also identified motifs enriched in candidate enhancers as compared to the 10 controls. Motif discovery was performed with STREME (--minw 6, otherwise default parameters) (Bailey, 2021)), also from MEME Suite. Discovered motifs were assessed for enrichment based on the same criteria described above (enriched relative to at least 8 of 10 control sets) using AME. To determine which of these discovered motifs were also present in JASPAR, the motifs were aligned with the 143 JASPAR motifs using TOMTOM (Gupta et al., 2007)), also from MEME Suite, using the default parameters.

Lastly, to determine if repetitive content differed between *de novo* discovered motifs as compared to those that align to JASPAR motifs, the nucleotide repeat content of the motif matches was compared. Repeats in the enhancers were defined and identified as described above and match coordinates were written to a BED file. The 36 discovered motifs were divided into the 22 that matched JASPAR motifs and the remaining 14 *de novo* motifs. The enhancer sequences were searched for motif matches using FIMO (Grant et al., 2011), from MEME Suite, and match coordinates were converted to BED files. Overlapping match regions in each BED file were merged using BEDtools (bedtools merge, default parameters). For each sequence, the number of nucleotides involved in a motif match and the number of nucleotides involved in both a motif match and a repeat match were counted and used to calculate the fraction of motif matched nucleotides that were repetitive per sequence.

### Comparison of Candidate STARR-Seq Enhancer Peaks With ChIP-Seq and ATAC-Seq Peaks

H3K27ac, H3K4me3, H3K9ac, and H3K9me3 ChIP-seq peak boundaries from uninfected *A. gambiae* samples were downloaded from Supplementary Table S3 of (Ruiz et al., 2019) and saved as four BED files containing the chromosome and peak position for each type of ChIP-seq experiment. ATAC-seq peaks for salivary glands and midguts from malaria-infected *A. gambiae* (Ruiz et al., 2021) were downloaded from NIH GEO repository GSE152924. The ATAC-seq peaks were separated by tissue and saved as BED files containing the chromosome, and peak position information. The ATAC-seq peaks were generated from multiple separate samples and contained overlapping peaks. Peaks from the two ATAC-seq data sets were merged using BEDTools (bedtools merge, default parameters), which reduced the number of peaks from 93,921 (midguts) and 99,084 (salivary glands) to 68,016 and 58,515, respectively. Coordinates of 20,578 computationally predicted enhancers were downloaded from Supplementary Table S1 of (Schember and Halfon, 2021) and saved as a single BED file. The coordinates were merged using BEDTools (bedtools merge, default parameters), resulting in 9,861 computationally predicted enhancer features. Overlaps between the ChIP-seq/ATAC-seq/computationally predicted peaks and STARR-seq peaks were performed using BEDTools (bedtools intersect -f 0.1 -r -u), which outputs each peak with 1 or more matches.

## Results

### Implementation of a Transcriptional Enhancer Screen in *Anopheles Coluzzii*

Methods developed for a massively parallel functional enhancer screen developed in *D. melanogaster* were modified and optimized for use in *A. coluzzii*. Efficiency of the screen was evaluated at multiple quality control steps to verify genome representation. First, the sheared and adaptor-ligated genomic DNA library was Illumina sequenced prior to cloning to measure genome coverage (Supplementary Figure S3A). Second, the library was Illumina sequenced after cloning into the STARR-seq reporter plasmid, but prior to transfection of 4a3A cells, to control for effects of cloning upon representative genome coverage (Supplementary Figure S3B), and sequenced again after transfection of three replicates and growth in 4a3A cells for 24 h, to control for culture effects on representation (Supplementary Figure S3C). The library retained high-quality genome coverage after each of these experimental steps. Median genome coverage was equivalent in the post-cloning sequence (median = 68) and the post-transfection sequences across three biological replicates (average median of 71, median range 66–78). Finally, the cDNA generated from the STARR-seq reporter plasmid displayed lower median genome coverage than the entire genome sequences above (Supplementary Figure S3D), which is consistent with the small fraction of the genome represented by enhancers. These sequencing controls indicate that the cloned reporter plasmid library of randomly-sheared *A. coluzzii* genomic fragments was representative and sufficiently powered to query the entire genome for detection of candidate enhancers.

### A Comprehensive Genome-Wide Map of Candidate Transcriptional Enhancers

**FIGURE 1:**
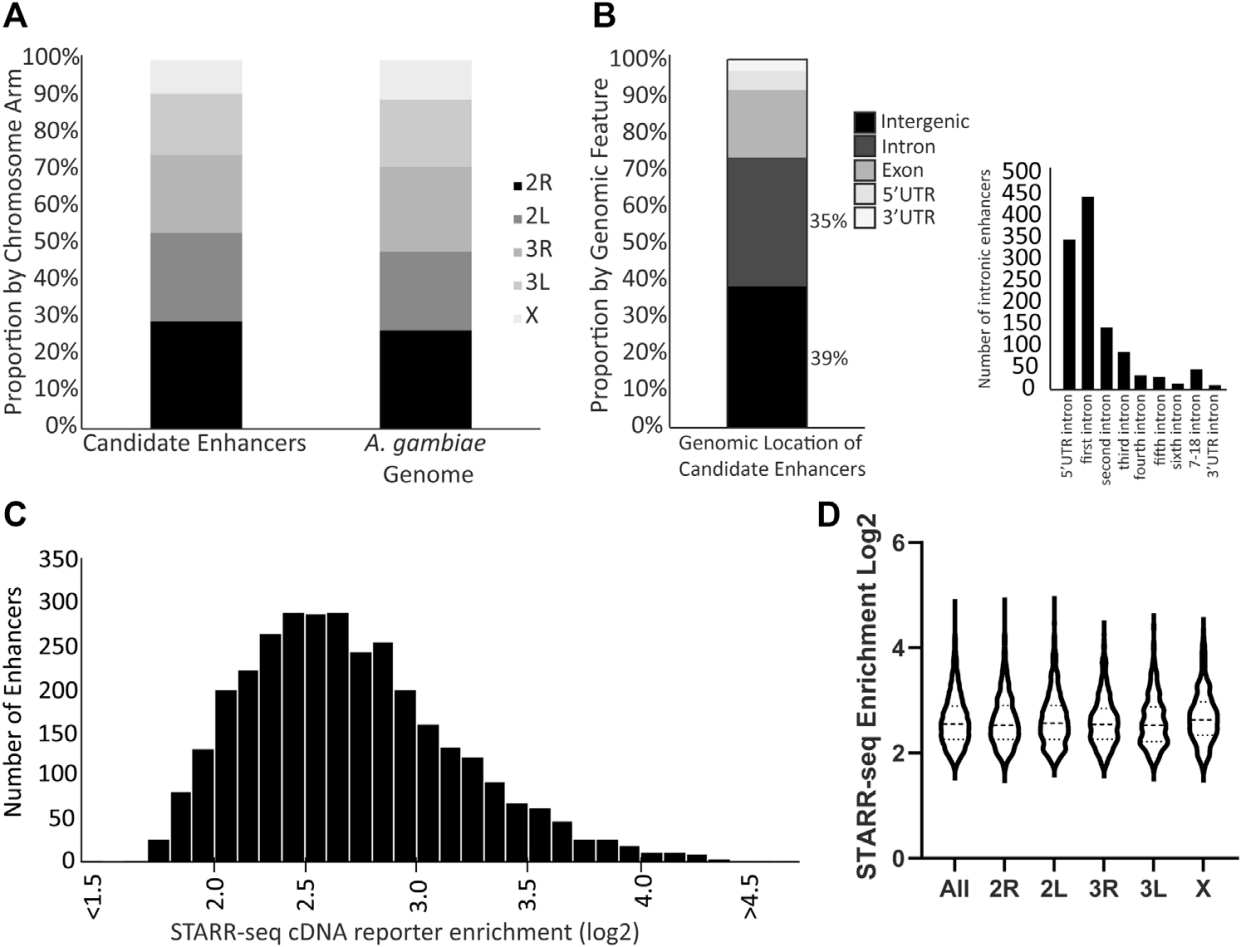
Genome distribution and activity of *A. coluzzii* candidate enhancers. **(A)** Candidate enhancers display a distribution across chromosome arms that equivalent to the total genome composition of chromosome arms. The *A. gambiae* reference genome assembly is used throughout because there is no chromosome-level assembly for *A. coluzzii*. **(B)** 74% of candidate enhancers are located in either intergenic or intronic regions. Of the 1,156 enhancers located in introns, nearly 70% are located in either the intron between the 5′UTR and the first coding exon (5′ UTR intron), or between the first and second coding exons (first intron). **(C)** Candidate enhancers vary in their activity levels as measured by the enrichment of reads of the enhancer sequence transcribed from the reporter plasmid (cDNA reporter) as compared to the same sequence in the control plasmid DNA samples. A log_2_ enrichment value of 2 represents a 4-fold increase in normalized read depth, log_2_ enrichment of 3 is an 8-fold increase and log_2_ enrichment of 4 a 16-fold increase. **(D)** Violin plot indicates that there is no significant difference in distribution of candidate enhancer strength across chromosome arms (middle horizontal lines represent median enrichment, ANOVA F = 1.631, *p* = 0.15).

Three biological replicates of the enhancer screen yielded a total of 3,288 candidate transcriptional enhancers present in all three replicates, hereafter referred to as the candidate enhancers. These candidate enhancers are located across the genome without enrichment on any particular chromosome arm (Figure 1A; chi-square = 1.170, df = 4, *p* = 0.88). The majority of candidate enhancers occur in either intergenic or intronic regions of the genome, with a small fraction overlapping exons (Figure 1B). A majority of the intronic candidate enhancers are found in the first intron (Figure 1B), similar to at least *D. melanogaster* (Arnold et al., 2013). The candidate enhancers vary in strength of activity, as measured by the normalized cDNA read depth values (Figure 1C), a distribution that does not differ by chromosome arm (Figure 1D). The complete catalog of candidate enhancers with position and read enrichment information is available in tabular format (Supplementary Table S2) as well as a graphic format (example in Supplementary Figure S4) generated using the Integrative Genomics Viewer tool (Robinson et al., 2011).

### Experimental Validation of Candidate Enhancers

Manual luciferase reporter assays were used to test a validation panel comprised of 28 candidate enhancers and 9 size-matched control fragments that did not display enhancer activity in the STARR-seq screen, and thus were predicted to not be enhancers. The candidates in the validation panel were selected solely on the criterion of location, with one candidate approximately each 10 MB across the genome (candidate coverage: chromosome X, 3; chromosome arm 2R, 7; chromosome arm 2L, 6; chromosome arm 3R, 7; chromosome arm 3L, 5). Control fragments were distributed to include one on chromosome X and two on each autosomal chromosome arm.

**FIGURE 2:**
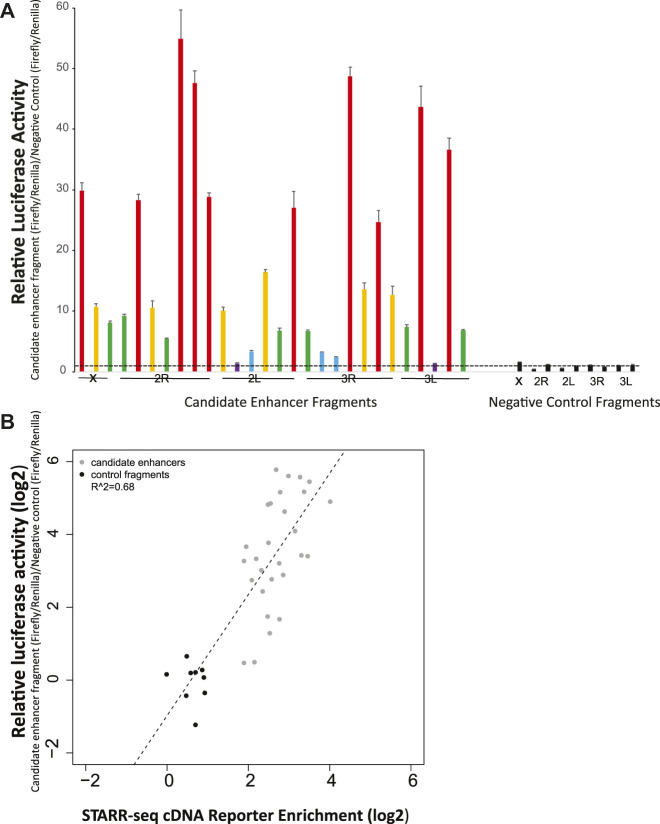
Experimental validation by manual luciferase reporter assays confirms accuracy of the genome-wide enhancer screen. **(A)** Twenty-eight candidate enhancers randomly chosen at ∼10 Mb spacing across the genome were tested for enhancer activity with a luciferase assay. Twenty six of the 28 displayed greater average activity than the nine negative controls, which were not predicted to be enhancer peaks in the genome-wide screen. None of the nine negative controls displayed measurable activity above background. Relatively luciferase activity was measured across two biological replicates, with six technical replicates per biological replicate. Relative activity of candidate enhancers is indicated by color: violet, relative luciferase activity <2, blue, activity 2-5, green, activity 5–10, yellow, activity 10–20, and red, activity >20. Negative control values are indicated in black. The horizontal dotted line indicates relative luciferase activity of 1, equal to that of the internal negative control used across all plates as previously (Nardini et al., 2019). **(B)** The correlation for validation panel enhancers between enrichment of cDNA reads in the genome-wide screen (*x*-axis) and relative luciferase activity in manual luciferase reporter assay (*y*-axis) indicates a significant relationship (*p* < 0.001) between the genome-wide screen and manual luciferase reporter assay, indicating the quantitative accuracy of the massively parallel screen for detection of relative activity levels of enhancers.

When tested by manual luciferase reporter assays, over ninety percent (93%, 26 of 28) of the tested candidate enhancers displayed higher average relative luciferase activity than any of the 9 tested negative controls (Figure 2A). All of the nine negative control fragments that were not enriched in the enhancer screen also did not display luciferase activity above background. These results indicate that the screen efficiently identified genome-wide enhancers in *A. coluzzii*. Researchers should specifically confirm specific candidate enhancers of interest before use.

The quantitative levels of enhancer activity of the validation panel, measured by manual luciferase reporter assays, were compared to the cDNA sequence enrichment values for the same enhancers as detected in the STARR-seq screen (Figure 2B). Manual luciferase activity values for an enhancer (*y*-axis, relative luciferase activity) were significantly correlated with STARR-seq cDNA enrichment values (*r* ^2^ = 0.68, *p* < 0.001). Enhancers displaying the greatest cDNA enrichment also display higher activity in the manual luciferase reporter assay. Thus, the enrichment measurements in the STARR-seq assay were not only a qualitative method suitable for identification of transcriptional enhancers, but were also accurate quantitative measurements of enhancer activity levels.

### Enhancer Activity in an Independent Cell Line

**FIGURE 3:**
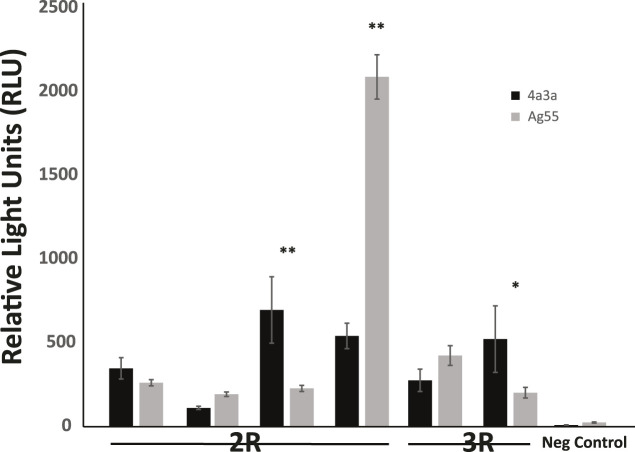
Enhancer activity is qualitatively consistent in independent cell lines, with quantitative differences. Activity of a panel of enhancers was measured in *A. coluzzii* cell lines 4a3A and Ag55 using dual glow luciferase assay with relative light units normalized to renilla. All fragments that displayed significant activity above baseline in 4a3A cells, also did so in Ag55 cells and the negative control fragment showed no activity in either cellular environment. Of the 6 tested enhancers, 3 displayed statistically indistinguishable activity in 4a3A and Ag55 cells, while the other 3 were differentially active (*p < 0.05, **p ≤ 0.001).

Enhancer activity for six candidate enhancers and one negative control was also tested in a second independently derived *A. coluzzii* cell line, Ag55. At a qualitative level, the results were independent of cell line, because fragments that displayed enhancer activity above background in 4a3A cells also did so in Ag55 cells. At a quantitative level, three enhancers showed statistically indistinguishable activity levels across cell lines, 2 enhancers had significantly higher activity in 4a3A cells and one enhancer displayed higher activity in Ag55 cells (Figure 3).

### Genome-wide Enhancer Catalog in Genome Browser Format

The list of the 3,288 detected candidate enhancer peaks is available in Supplementary Table S2. BED files (Datasheet 5 & Datasheet 6) are also provided that allow the visualization of detected peaks alongside the gene models currently available for the PEST genome assembly of *A. gambiae.* To visualize and search the genome wide map of enhancers open the above BED files (Supplementary Figure S4) in Integrative Genomics Viewer (Robinson et al., 2011).

### Comparative Sequence Analysis of Enhancers and Non-Coding Control Fragments

The 3,288 functionally detected enhancers from the screen together occupy a total of approximately 1.85 Mb, or 0.7% of the *A. coluzzii* 281.38 Mb genome. We evaluated the enhancer compartment in comparison to matched genomic controls in order to identify distinct composition features and sequence patterns of the enhancer compartment of the genome that would be expected to underlie the functional properties of the *A. coluzzii* enhancers. The controls were ten independent genomic control sets extracted from the fraction of the genome that did not display enhancer activity peaks in the genome-wide screen. The control sets were matched to the enhancer compartment for equivalent fragment size and total size as the summed genomic enhancers.

GC Content Analysis. The enhancer sequence compartment displayed significantly higher GC content than the non-enhancer controls (Supplementary Figure S5, enhancer compartment 49.9 ± 4.5%, control set 43.8 ± 6.8%, Mann-Whitney U *p*-value = 1.6 × 10^−304^). In addition to being more GC-rich relative to control sequences, the enhancer compartment displayed smaller variation in GC across the set of candidate enhancers.

**FIGURE 4:**
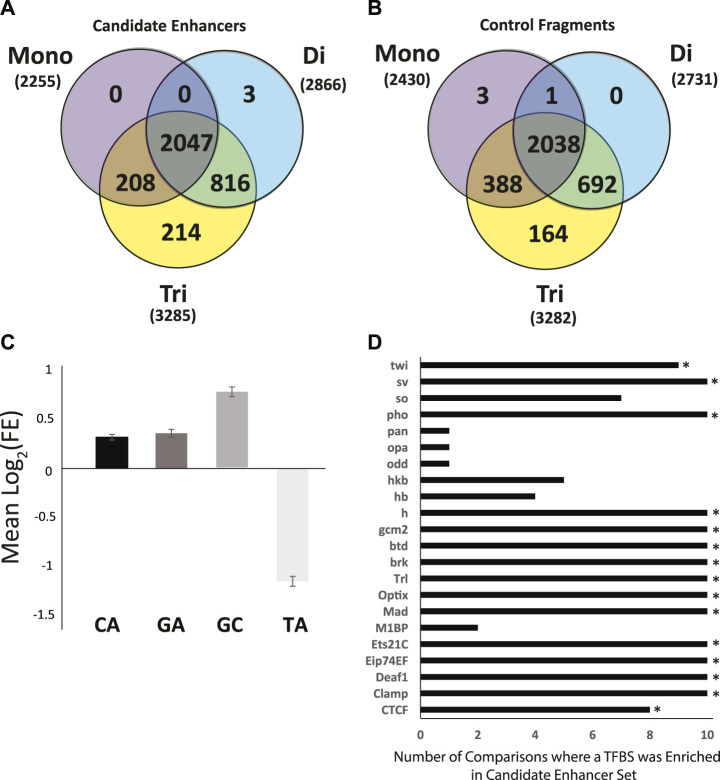
Sequence characteristics of the *Anopheles coluzzii* enhancer compartment as compared to control sequences. The enhancer compartment represents ∼0.7% of the total genome sequence. **(A)** Venn diagram of the number of candidate enhancers that contain perfect mono, di and tri nucleotide repeats, defined as sequences ≥6 bp in length (e.g., AAAAAA, ATATAT, ATCATC). **(B)** As in **(A)**, but for 3288 control fragments not detected as enhancers by the genome-wide screen (two control sequences lacked any repetitive sequence meeting the definition described and are not displayed). **(C)** Mean log_2_ of the fold-enrichment (FE) in perfect dinucleotide repeats between candidate enhancer sequences and controls. Numbers greater than 0 indicate enrichment in candidate enhancer sequences and numbers less than 0 indicate enrichment in control sequences. **(D)** Sequences of candidate enhancers and controls were searched for predicted transcription factor binding sites (TFBS) using AME from MEME Suite and enrichment of computationally predicted TFBS was computed. Graph indicates the number of control data sets where the TFBS was depleted as compared to candidate enhancers. Fifteen TFBS (indicated by *) were enriched in the candidate enhancer sequences above the defined threshold (enhancer sequences were tested against each of 10 control sequence sets for a total of 10 runs, threshold for motif enrichment was a positive test in at least 8 of 10 runs).

Perfect Repeat Analysis. Perfect mononucleotide, dinucleotide, and trinucleotide repeats of 6 or more nucleotides in length were analyzed. As a whole, there were no significant differences in the types of repeats found in candidate enhancer sequences as compared to genome matched control sequences (Figures 4A,B). There were, however, specific repeats that were over- or under -represented amongst enhancer sequences as compared to controls. Specifically, repeats rich in A or T (A, T, AT, TA, AAT, ATA, ATT, TAA, TAT, TTA) were significantly enriched in all control data sets in terms of repeat presence. Conversely, those rich in G and C (AC, CA, CG, CT, GC, GT, TG, ACG, AGC, CAC, CAG, CCG, CGA, CGC, CGG, CGT, CTG, GCA, GCC, GCG, GCT, GGA, GGC, GGT, GTG, TCC, TCG, TGC) were significantly enriched in the enhancer sequences. Enrichment in GC rich repeats is consistent with the difference in overall GC content presented above.

Enrichment of CA, GA, GC, and TA dinucleotide repeats was analyzed in candidate enhancer sequences. *A. coluzzii* enhancers detected in the current screen displayed an enrichment of CA, GA, and GC repeats and depletion of TA (The GA enrichment in candidate enhancer fragments was statistically significant in 8 of 10 post-hoc tests but did not meet the criteria used above for enrichment). GC was enriched at two times the rate of GC or CA (Figure 4C). The CA repeat was present in 42.4% of enhancer sequences compared to only 33.9 ± 0.7% of controls (*p* = 6.3^∗^10^−58^). For the GA repeat, these numbers were 20.0% in candidate enhancer sequences and 15.6 ± 0.4% in controls (*p* = 2.9^∗^10^−26^) and for GC, 21.8% in candidate enhancers and 12.8 ± 0.5% in controls (*p* = 2.4^∗^10^−108^). Conversely with the TA repeat, only 4.7% of enhancer sequences contained this sequence while 10.5 ± 0.4% of control sequences did (*p* = 3.4^∗^10^−65^).

Transcription Factor Binding Sites (TFBS) Definitions of 143 TFBS from the JASPAR CORE 2020 insect release were tested for enrichment in the enhancer compartment as compared to genome matched controls. Each definition was tested in 10 iterations, once against each control. TFBS that were enriched in at least 8 of 10 tests were deemed enriched. In total, 15 of the 143 TFBS tested were enriched in candidate enhancer sequences as compared to control sequences by our criteria (Figure 4D).

In addition to searching against the TFBS annotated in JASPAR, motif discovery was also performed. A total of 36 motifs were discovered, all of which were enriched according to our criteria (Supplementary Table S3). Twenty-two of the 36 discovered motifs aligned with one or more of 143 JASPAR motifs, including all 15 JASPAR motifs found enriched in the above analysis. Fourteen of the discovered motifs did not match any of the 143 JASPAR motifs and potentially represent uncharacterized TFBS active in the 4a3A cells. The *de novo* motif content was more repetitive (35.6 ± 24.9%; Rank Sums test, *p* = 2.2^∗^10^−37^) than the JASPAR motif content (27.9 ± 17.8%), suggesting that not all enriched *de novo* motifs may be functional binding sites given the lack of complexity of the highly-repetitive sequence (Supplementary Figure S6), but may serve other functions that otherwise influence enhancer activity.

### Comparison of Candidate Enhancers With ChIP-Seq, ATAC-Seq and Computationally Predicted Peaks

The *A. coluzzii* candidate enhancers detected by STARR-seq were compared with peaks from published ChIP-seq and ATAC-seq analyses in *A. gambiae* and with computational enhancer predictions (Supplementary Table S4). The ChIP-seq data detected four histone modifications (H3K27ac, H3K9ac, H3K9me3 and H3K4me3) associated with enhancers, promoters, and gene activation and gene silencing, respectively, in a comparison of mosquitoes infected with the malaria parasite *Plasmodium falciparum* or uninfected controls (Ruiz et al., 2019). The largest overlap with STARR-seq candidate enhancers occurred with the 19,321 H3K27ac ChIP-seq peaks, which are indicative of active enhancers and promoters, because 22.8% of the 3,288 *A. coluzzii* STARR-seq peaks overlapped with the H3K27ac ChIP-seq peaks. STARR-seq candidate enhancer peaks were also compared with data collected by ATAC-seq on midguts and salivary glands from malaria-infected *A. gambiae* samples (Ruiz et al., 2021). Of the 3,288 *A. coluzzii* STARR-seq peaks, 33.6 and 35.3% overlapped with ATAC-seq peaks identified from midguts and salivary gland samples, respectively. Schember and Halfon (Schember and Halfon, 2021) performed *in silico* computational prediction of enhancers with training sets derived from *D. melanogaster* and organized by active tissues. After merging, 9,861 unique peaks were computationally predicted to be enhancers, and 6.8% of the STARR-seq candidate enhancer peaks overlapped these predicted enhancers. Across all of the data sets, most of the overlaps were one-to-one, because a single STARR-seq peak overlapped with a single peak identified by another method. The partial overlap between the different data sets may tend to support the candidate enhancers identified by STARR-seq, but further work will be required to determine the proportion of ChIP-seq and ATAC-seq peaks that represent functional enhancers, as compared to other categories of chromatin features. Indeed, the STARR-seq data could serve as a training set to refine computational models, which are built upon training data.

## Discussion

We present a genome-wide map of 3,288 transcriptional enhancers identified by screening in wild samples of the African malaria vector mosquito, *A. coluzzii*. This work generates a resource that will help make the functional non-coding regulatory DNA accessible to study in this mosquito. Manual testing of a validation panel from the genome-wide catalog demonstrated the high accuracy of the screen for enhancer detection, as well as the agreement of quantitative enhancer activity levels between the highly multiplexed genome-wide screen and values obtained for the same enhancers from individual manual tests. The identified enhancers in *A. coluzzii* display transcriptional enhancing activity levels over a wide dynamic range of at least two-fold above baseline and up to at least 16-fold higher activity. These results demonstrate that genomic regions with significant transcriptional regulatory potential were detected and that the quantitative measurement of their activities by the genome-wide screen are likely to correspond to their biological activity levels *in vivo*.

The two methods used in this study for enhancer detection and quantitation, cDNA enrichment in STARR-seq or manual luciferase reporter assays, share the property that, as ectopic plasmid-based assays, they measure the innate sequence-based enhancer activity of the candidate fragment within the cellular environment, but without the influence of chromatin and its chemical modifications. The ectopic plasmids carrying the candidate genome fragments are not chromatinized, while enhancers in the chromosomal environment are modulated by natural chromatin. Thus, the genome-wide enhancers detected in the current study represent a catalog of genomic enhancers expected to be active in diverse cell types or developmental stages of the organism in which the same transcription factors are active, while only a subset of enhancers are expected to be biologically active in a specific cell type and/or developmental stage. Cross validation of a small panel of enhancers in a second *A coluzzii* cell line indicates that the tested enhancers were active in both cell lines. This observation suggests that, at least for this test panel, the cellular machinery of transcription factors and binding partners for transcriptional regulation is comparable among two independently-derived *A. coluzzii* cell lines from the same tissue origin. Three of the six enhancers tested displayed different levels of positive activity among the 2 cell lines. The most likely explanation is that there are abundance differences in the cell lines of transcription factors and partners relevant to each specific enhancer. However, the importance of the activity differences for natural mosquito biology, if any, should be interpreted with caution. The activity differences between cell lines could result from capturing natural allelic variation for transcription factor abundance in the 2 cell lines, which would be interesting, but could also be an *in vitro* artifact caused by genetic drift and/or slightly divergent adaptation of the 2 cell lines to the culture environment. Enhancers with cell-type or developmentally restricted activity result from the different combinations of transcription factors expressed in a given cell type (Kheradpour et al., 2013). Biologically active enhancers are found in open chromatin, which exposes them to binding by transcription factors and other factors, while closed chromatin obscures the enhancers that are not active in the specific cell such that they are not accessible for transcription factor binding.

The subset of enhancers that are biologically active in a given cell can only be detected by indirect means in nuclei isolated from a pure sample of the target cell type or stage. Previous work on mosquito non-coding regulatory elements has employed chromatin-based approaches (Behura et al., 2016; Perez-Zamorano et al., 2017; Mysore et al., 2018; Ruiz et al., 2019; Lezcano et al., 2020; Nowling et al., 2021; Ruiz et al., 2021). However, for phenotypic studies, pure samples of the relevant cell type may not be feasible to obtain. Moreover, during initial exploration of a phenotype, the relevant cell type or timing may not be known, or multiple sites may be responsible. Thus, use of the current comprehensive catalog of *A. coluzzii* enhancers in phenotype studies can be combined with subsequent chromatin-based cell-specific assays once the target cell type is known, in order to clearly define the genome regulatory space underlying the phenotype. Previous work has focused on computational prediction of enhancers in *Anopheles* (Schember and Halfon, 2021) and in highly diverged insects (Kazemian et al., 2014). Integration of the current genome-wide comprehensive catalog with chromatin-based cell and developmental data can be used to train algorithms which may be able to more efficiently predict transcriptional enhancers from sequence context.

STARR-seq is a method that determines and quantifies the ability of a DNA sequence to enhance transcription in a chromatin-independent episomal context, while ChIP-seq and ATAC-seq determine biologically active chromatin features, including but not specifically limited to enhancers. We demonstrated a degree of overlap of candidate enhancers functionally detected using STARR-seq with *Anopheles* chromatin features. Almost all (93%) of the STARR-seq enhancer peaks displayed enhancer activity by manual standard luciferase assays, while just 26% of ENCODE enhancer predictions made using ChIP-seq displayed regulatory activity (Kwasnieski et al., 2014). Thus, chromatin modifications have the potential to indicate enhancer sequences in the genome, but direct activity assays that measure transcriptional enhancing activity from candidate containing TFBS sequences are more sensitive and precise for identifying enhancers. The combination of functional assays such as STARR-seq coupled with chromatin-based methods will be essential in the dissection of regulated gene expression.

Computational analyses of the current enhancer catalog revealed relative enrichment and depletion of particular dinucleotide repeats within the enhancer space of the *A. coluzzii* genome. Similar patterns of repetitive sequence enrichment and depletion have also been demonstrated in humans and *D. melanogaster*. The functional role or importance of these repeats is not yet clearly demonstrated in any organism, but repeated discovery of the same pattern across invertebrates and vertebrates suggests a level of evolutionary functional conservation (Andolfatto, 2005; Cande et al., 2009; Glassford and Rebeiz, 2013; Schwaiger et al., 2014; Villar et al., 2015). The simplest interpretation is that the nucleotide composition and dinucleotide repeat enrichment or depletion in the enhancer compartment of the genome is a consequence of functional constraints imposed by binding of proteins such as transcription factors to TFBS, which underlies enhancer function as currently understood (Yao et al., 2015; Jacobs et al., 2018; Lambert et al., 2019). Fifteen motifs defining insect TFBS from the *D. melanogaster* JASPAR database and an addition 14 *de novo* motifs were significantly enriched in the *A. coluzzii* enhancer compartment. Further work will be necessary to confirm which of these potential TFBS are functional in enhancers of *A. coluzzii*, because *Anopheles* transcription factors and TFBS have not yet been comprehensively characterized. Thirteen of the fifteen putative transcription factors enriched in STARR-seq candidate enhancers (Figure 4D, TF identities inferred from the TFBS) have orthologues in both *A. coluzzii* and *A. gambiae*. Twelve of these 13 are one-to-one orthologues among the two anophelines, and the other is a *D. melanogaster* TF that matches two predicted proteins in both *A. coluzzii* and *A. gambiae* (Supplementary Table S5). The evolutionary conservation supports the conclusion that these TFs and their cognate binding sites are likely to be functional in *A. coluzzii*.

Human genetic mapping data suggest that non-coding variation is responsible for the majority of phenotypic variation. Of the significant SNPs associated with phenotypes in genome-wide association, studies (GWAS), only 5–10% are protein-coding variants, and >90% of significant GWAS hits are non-coding SNPs (Neph et al., 2012; Schaub et al., 2012; Farh et al., 2015). Mutations in human enhancers are associated with risk states for arthritis and growth disorders (Capellini et al., 2017), neurodevelopmental disorders (Short et al., 2018) and susceptibility to infection (Telenti and di Iulio, 2020), among others. In yeast, variable non-coding regions contribute to phenotypic diversity (Salinas et al., 2016), and a transcribed enhancer in *C. elegans* regulates the development of egg laying muscles (Goh and Inoue, 2018). In a recent association mapping study of *Anopheles* desiccation resistance, most of the significant SNPs were located in non-coding regions (Ayala et al., 2019), and therefore could be located in regulatory elements. The enhancer catalog presented here will be a useful tool to help decode the phenotypic effects of polymorphic non-coding DNA in *Anopheles*, particularly the important *Anopheles* traits underlying malaria transmission and vector biology such as *Plasmodium* infection susceptibility, insecticide resistance, and others.

However, understanding phenotypic readouts of enhancer function, or the differential phenotypes caused by allelic enhancers, is complicated by the incomplete current understanding of the mechanism of enhancer function. Most importantly, enhancers function by *cis*-activating the promoters of a panel of target genes that can be located tens or hundreds of kilobases distant, and the regulated target genes are often not the nearest gene(s) to the enhancer. Similar to the inability to accurately predict enhancer location computationally due the absence of a defined sequence code, there is also no current capacity to computationally predict the target genes of an enhancer. Target gene detection and modeling is most advanced in the human system, where a combination of empirical chromatin-based assays and computational modeling of interaction networks has indicated that most enhancers regulate up to approximately ten target genes, but that a given gene can be regulated by multiple enhancers with presumably contrasting effects (Mumbach et al., 2017; Baxter et al., 2018; Gasperini et al., 2020; Reilly et al., 2021). Thus, understanding the phenotypic outcomes of enhancer regulatory function is still in the early stages even in the most characterized model organisms.

The current genome-wide catalogue of transcriptional enhancers will provide a useful tool to identify enhancer locations, understand the important drivers of gene expression, and functionally filter the effects of genetic variation in *Anopheles* genotype-phenotype studies. Knowledge of the genomic locations of enhancers can also aid in the design of CRISPR/Cas9-based genome editing experiments, because chromosomal enhancers could alter the behavior of a gene integrated nearby, or an unknown enhancer included in an integrated cassette could produce unexpected expression results. Overall, the current catalog of *cis*-regulatory enhancer elements will contribute to developing a more complete picture of *cis*-regulatory modules within *Anopheles*, and provides a new tool for biological investigation aimed at the design of improved vector control methods.

## Data Availability

All sequence files are available from the EBI European Nucleotide Archive database (http://www.ebi.ac.uk/ena/) under ENA study accession number PRJEB34434. The genome wide enhancer catalog generated in this study is available in Supplementary Table S2.
