# Supplementary figures and images for: Comprehensive Genomic Discovery of Non-Coding Transcriptional Enhancers in the African Malaria Vector Anopheles coluzzii
Source: Front Genet. 2022 Jan 10;12:785934. doi: 10.3389/fgene.2021.785934 (PMC8784733; doi:10.3389/fgene.2021.785934)

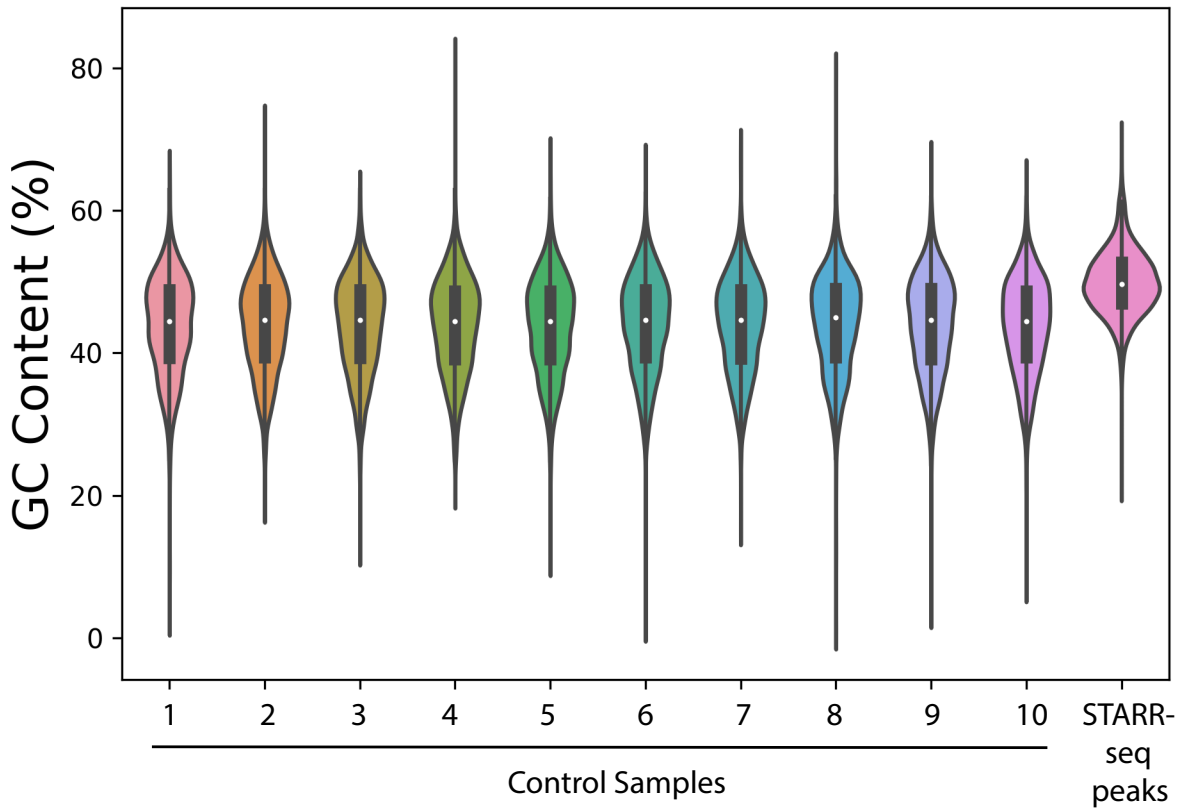

Supplement: Supplementary file 1 [file DataSheet7.PDF]

Acol\_4A3A\_repbio1

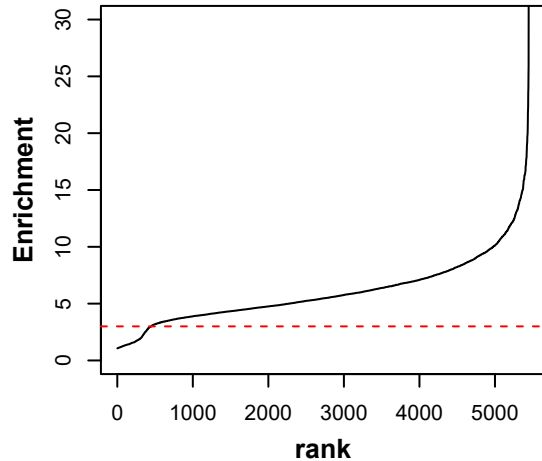

Acol\_4A3A\_repbio2

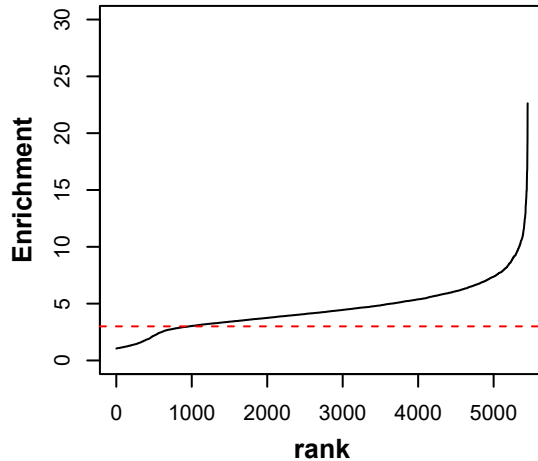

Acol\_4A3A\_repbio3

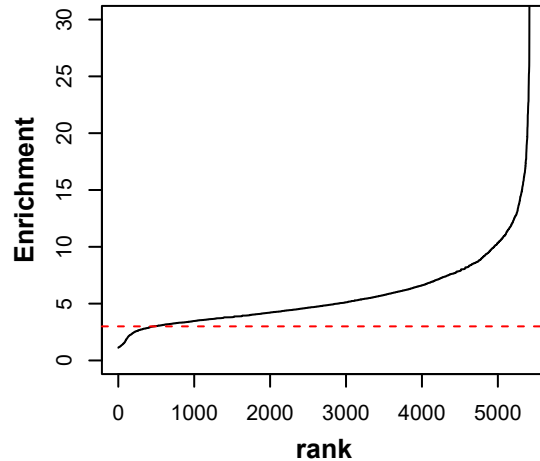

Supplement: Supplementary file 3 [file DataSheet4.PDF]

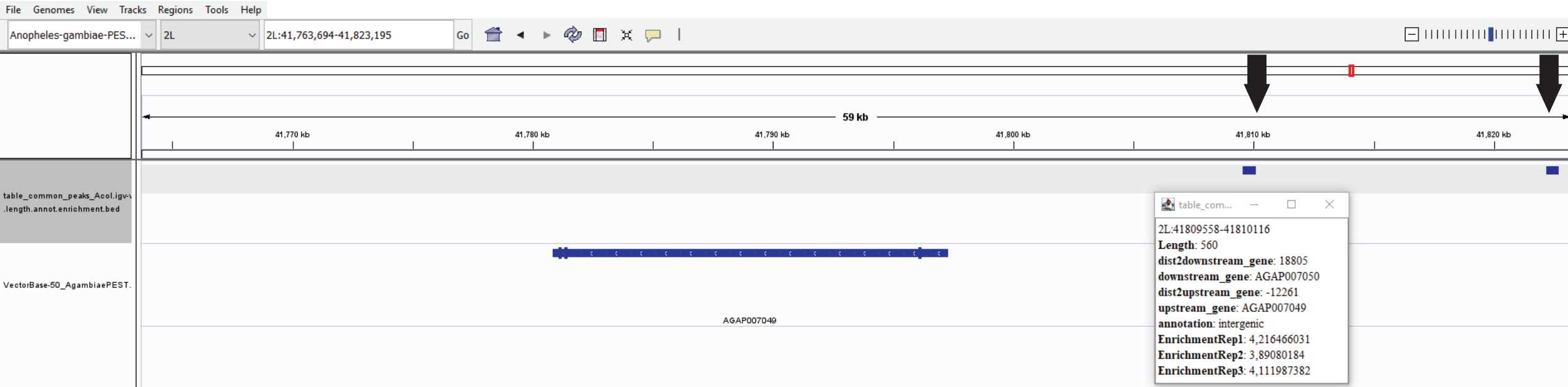

Supplement: Supplementary file 4 [file DataSheet6.PDF]

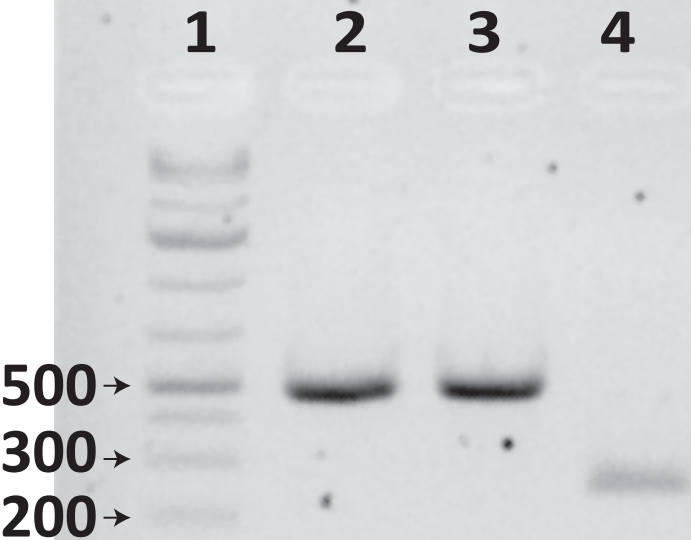

Supplement: Supplementary file 7 [file DataSheet3.PDF]

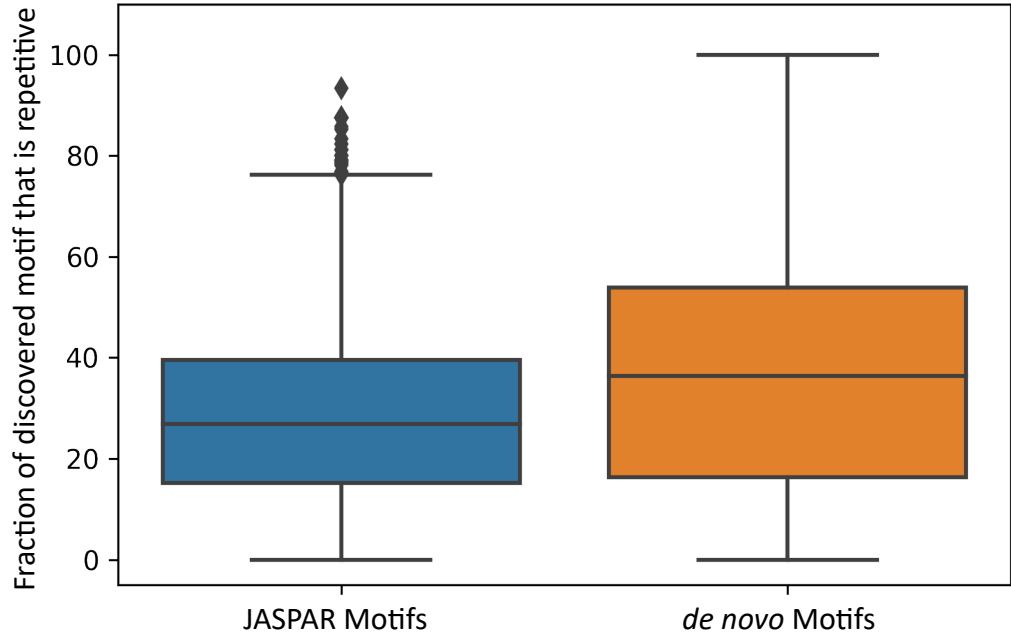

Supplement: Supplementary file 12 [file DataSheet8.PDF]
