# Supplementary material for: Comprehensive Genomic Discovery of Non-Coding Transcriptional Enhancers in the African Malaria Vector Anopheles coluzzii
Source: Front Genet. 2022 Jan 10;12:785934. doi: 10.3389/fgene.2021.785934 (PMC8784733; doi:10.3389/fgene.2021.785934)
Supplement: Supplementary file 8 [file DataSheet5.PDF]

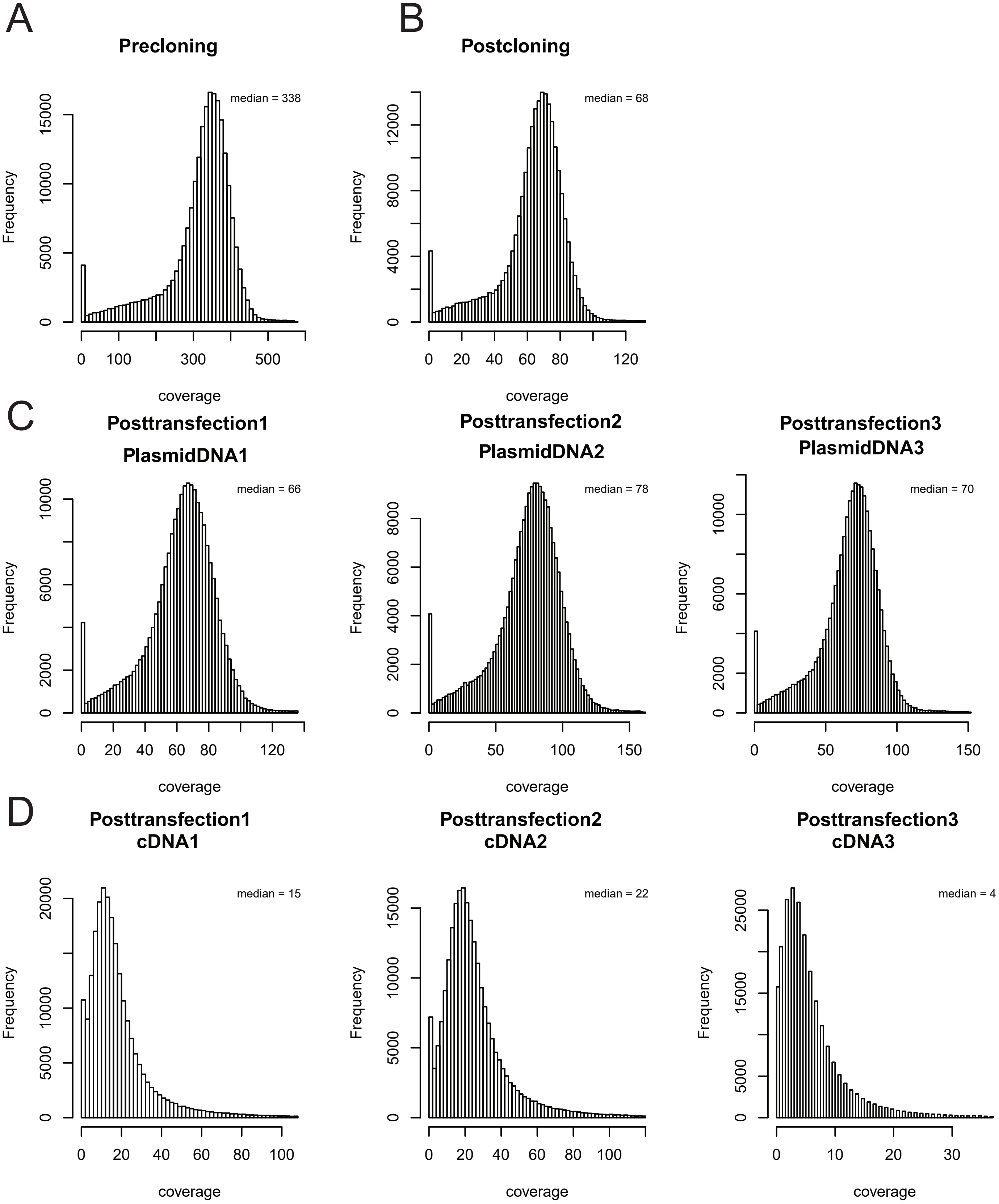
A
Precloning
median = 338
Frequency
coverage
B
Postcloning
median = 68
Frequency
coverage
C
Posttransfection1
PlasmidDNA1
median = 66
Posttransfection2
PlasmidDNA2
median = 78
Posttransfection3
PlasmidDNA3
median = 70
Frequency
coverage
D
Posttransfection1
cDNA1
median = 15
Posttransfection2
cDNA2
median = 22
Posttransfection3
cDNA3
median = 4
Frequency
coverage
